# Mg-LDH Nanoclays Intercalated Fennel and Green Tea Active Ingredient: Field and Laboratory Evaluation of Insecticidal Activities against *Culex pipiens* and Their Non-Target Organisms

**DOI:** 10.3390/molecules27082424

**Published:** 2022-04-08

**Authors:** Ibrahim Taha Radwan, Mohamed M. Baz, Hanem Khater, Abeer Mousa Alkhaibari, Abdelfattah M. Selim

**Affiliations:** 1 Supplementary General Sciences Department, Faculty of Oral and Dental Medicine, Future University in Egypt, Cairo 11835, Egypt; ibrahim80radwan@hotmail.com; 2 Department of Entomology, Faculty of Science, Benha University, Benha 13518, Egypt; mohamed.albaz@fsc.bu.edu.eg; 3 Department of Parasitology, Faculty of Veterinary Medicine, Benha University, Toukh 13736, Egypt; hanemkhater@gmail.com; 4 Department of Biology, Faculty of Science, University of Tabuk, Tabuk 71491, Saudi Arabia; aalkhaibari@ut.edu.sa; 5 Department of Animal Medicine (Infectious Diseases), College of Veterinary Medicine, Benha University, Toukh 13736, Egypt

**Keywords:** layered double hydroxides, nanoclays, Mg LDH, Ni LDH, *Foeniculum vulgare*, *Camellia sinensis*, non-target organisms, predation

## Abstract

(1) Background: Mosquito control with essential oils is a growing demand. This work evaluated the novel larvicidal and adulticidal activity of fennel and green tea oils and their Layered double hydroxides (LDHs) nanohybrid against *Culex pipiens* (*Cx. pipiens*) in both laboratory and field conditions and evaluated their effect against non-target organisms; (2) Methods: Two types of nanoclays, MgAl-LDH and NiAl-LDH were synthesized and characterized using PXRD, TEM and SEM, whereas their elemental analysis was accomplished by SEM-EDX; (3) Results: Mg and Ni LDHs were synthesized by the co-precipitation method. The adsorption and desorption of active ingredients were conducted using LC MS/MS, with reference to the SEM-EXD analysis. The desorption process of MgAl-LDH intercalated green tea oil was conducted using ethanol, and reveled significant peaks related to polyphenols and flavonoids like Vanillin, Catechin, Daidzein, Ellagic acid, Naringenin, Myricetin and Syringic acid with concentrations of 0.76, 0.73, 0.67, 0.59, 0.52, 0.44 and 0.42 μg/g, respectively. The larvicidal LC_50_ values of fennel oil, Mg-LDH-F, and Ni-LDH-F were 843.88, 451.95, 550.12 ppm, respectively, whereas the corresponding values of green tea were 938.93, 530.46, and 769.94 ppm. The larval reduction percentage of fennel oil and Mg-LDH-F reached 90.1 and 96.2%, 24 h PT and their persistence reached five and seven days PT, respectively. The reduction percentage of green tea oil and Mg-LDH-GT reached 88.00 and 92.01%, 24 h PT and their persistence reached five and six days PT, respectively. Against adults, Mg-LDH-GT and Ni-LDH-GT were less effective than green tea oil as their LC_95_ values were 5.45, 25.90, and 35.39%, respectively. The reduction in adult density PT with fennel oil, Mg-LDH-F, green tea oil, and Mg-LDH-GT reached 83.1, 100, 77.0, and 99.0%, respectively, 24 h PT and were effective for three days. Mg-LDH-GT and Mg-LDH-F increased the predation *Cybister tripunctatus* (71% and 69%), respectively; (4) Conclusions: For the first time, Mg-LDH-GT and Mg-LDH-F was the best system loaded with relatively good desorption release to its active ingredients and significantly affected *Cx. pipiens* larvae and adults in both laboratory and field circumstances, and it could be included in mosquito control.

## 1. Introduction

**Figure 1:**
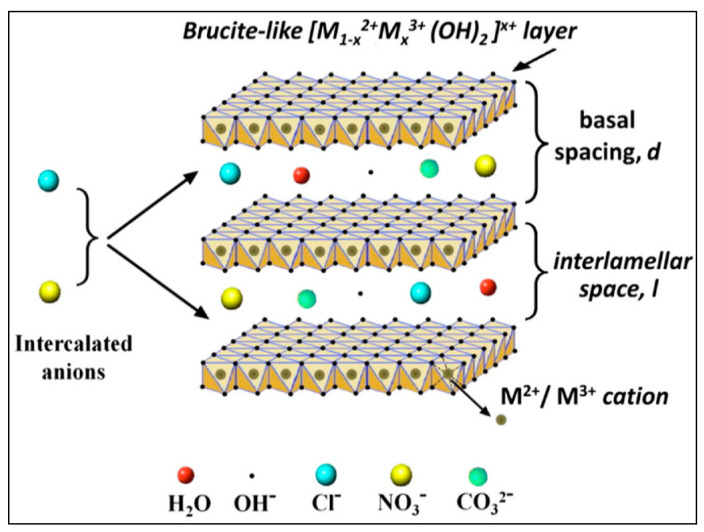
Three dimensions tunable LDH interlayer structure [20].

Hydrotalcite and Hydrotalcite-Like Layered double hydroxides (LDHs) nano sheets have attracted significant attention due to their potential application in different domains of photochemistry, catalysis, polymerization, electrochemistry, environmental science, and biomedical applications [1,2,3]. LDHs are synthetic anionic nanoclays or hydrotalcite-like structures, consisting of positively charged metal oxide and sheets of hydroxide with intercalated anions and water molecules. For MgAl-LDH, stacked layers of brucite [Mg(OH)_2_] have some of its divalent cations as Mg^2+^ substituted by trivalent cations Al^3+^ at the centers of the octahedral sites of the hydroxide sheet, in which the vertex contains hydroxide anions that are distributed by three octahedral cations and pointed toward the interlayer region. They are represented by the formula: [M^2+^_(1−x)_ + M^3+^_x_ (OH)_2_]^X+^ (A^n−^)_x/n_^x−^·mH_2_O, where M^2+^ and M^3+^ is a divalent cation such as Mg^2+^, Zn^2+^, Ni^2+^ and trivalent cation such as Al^3+^, Cr^3+^ and Fe^3+^, respectively. Owing to the partial substitutions of M^3+^ for M^2+^, the LDHs sheets are positively charged and need to be neutralized by the intercalation of anions (A^n−^), like NO_3_^−^, Cl^−^, CO_3_^2−^ or SO_4_^2−^ whereas, the x value is calculated by the ratio MIII/MII +MIII and is usually between 0.17 and 0.33, however, higher x values have also been reported [4,5]. LDHs possess a well-defined layered structure (Figure 1) with unique properties. The tunable composition of LDH nanoclays are not the only the result of the divalent or trivalent cations being replaced by other cations, as the interlayer anion is easily replaced by another one and, consequently, their chemical and physical properties will be altered [6]. LDHs exhibit unique adsorption characteristics due to their large surface area, thermal stabilities, high adsorption and desorption [7,8,9]. Such materials have been widely used as adsorbents for gas molecules [10,11,12,13] and active ingredients or ions [14,15,16], and catalysts [17,18,19].

Mosquitoes are serious pests and the most important vectors of human disease as malaria, yellow, encephalitis, West Nile virus, yellow fever and filarial nematodes; therefore, mosquito control is a critical demand worldwide, particularly in the tropics and warm areas [21]. *Culex pipiens* (Diptera: Culicidae) is a nuisance mosquito distributed in Egypt and worldwide, transmitting diseases to humans and animals [22,23,24].

Synthetic insecticides and repellents are widely used to control pests, however their overuse, and misuse, resulted in pest resistance, environmental pollution, and health risks to humans and non-target organisms. Consequently, the discovery of natural alternatives to dangerous synthetic pesticides has drawn medical and economic attention worldwide due to their biodegradability, low toxicity, and ability to overcome insecticide resistance [25,26,27].

Essential oils (EOs) are increasingly popular among organic producers and ecologically concerned consumers due to their acceptability in urban environments, homes, and other sensitive locations. Monoterpenes, biogenetically related phenols, and sesquiterpenes are the main components of essential oils [27]. Effectively EOs were used for insect control [28,29,30].

Green tea leaves contain numerous polyphenols, particularly catechins, which make about 30–40% of the extractable solids in dried green tea leaves [31,32]. Besides polyphenols, it also contains proanthocyanidins (tannins), which are one of the allelochemicals produced by *Camellia sinensis* against insects [33]. Anti-cancer, antibacterial, antiviral, nematocidal, anti-allergic, cardioprotective, and cholesterol-lowering properties are all found in proanthocyanidins, whereas the ingestion of proanthocyanidins through green tea leaves causes harmful effects on the insects by attacking the midgut after breaking them down to free radicals [34]. *Foeniculum vulgare* oil contains many bioactive terpenoids, flavonoids, steroids, saponins, and tannins that play a role in the death of many insects including *Culex quinquefasciatus*, *Aedes aegypti*, and *Anopheles stephensi* mosquitoes [35,36].

This study aimed to evaluate the larvicidal and adulticidal effects of fennel and green tea oils and their Mg and Ni- LDH Nanoclays against *Cx. pipiens* in vitro and field evaluations, besides testing their efficacy against non-target predators for the first time.

## 2. Materials and Methods

### 2.1. Chemistry

#### 2.1.1. Materials

Magnesium Nitrate hexahydraes Mg(NO_3_)_2_·6H_2_O, Nickel nitrate hexahydrate Ni (NO_3_)_2_·6H_2_O, Aluminum Nitrate nonahydrates Al(NO_3_)_3_·9H_2_O, Sodium Hydroxide NaOH, hydrolyzed ammonium hydroxide (50%) and decarbonated water purchased from Alfa Aesar, Germany. Two essential oils, *Foeniculum vulgare* and *Camellia sinensis* were purchased from the EL CAPTAIN Company for extracting natural oils, plants, and cosmetics “Cap Pharm”, El Obor, Cairo, Egypt. All chemicals were used without further purification. 

#### 2.1.2. Synthesis of Mg and Ni Al-LDHs

**Figure 2:**
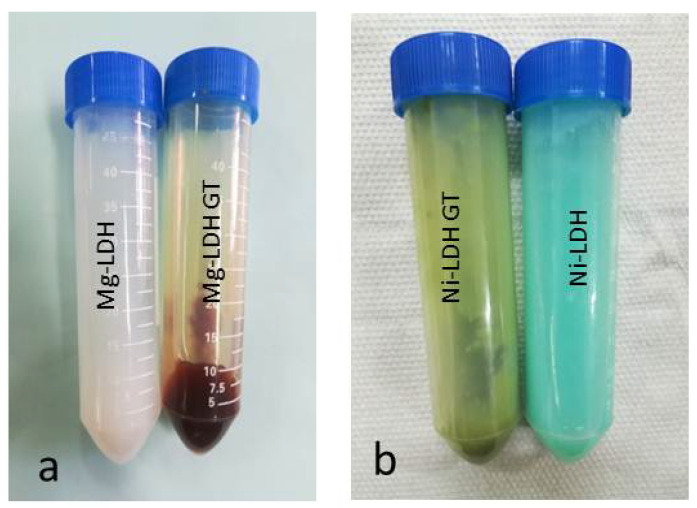
(**a**) MgAl-LDH and MgAl-LDH loaded green tea, (**b**) NiAl-LDH and NiAl-LDH intercalated green tea interlayer structure.

A mixture of Mg(NO_3_)_2_·6H_2_O (0.5 M) and Al(NO_3_)_3_·9H_2_O (0.25 M) with [Mg^2+^]/[Al^3+^] (2:1) molar ratio was dissolved in 50 mL decarbonated water and placed in a separatory funnel fitted in one gas inlet-outlet three-neck flask RBF apparatus. Then, 100 mL of NH_4_OH (35%) was prepared separately and placed in the second neck of the gas inlet-outlet RBF apparatus. Separately before the addition, both solutions were degassed by purging purified nitrogen gas for 10 min. The mixed salt solution of Mg(NO_3_)_2_·6H_2_O and Al(NO_3_)_3_·9H_2_O in the separatory funnel was added dropwise to the solution of NH_4_OH, with vigorous stirring (1500 rpm) at room temperature under blanket of nitrogen by passing nitrogen gas throw the gas inlet-outlet RBF. The Stirring continued for 2 h and the pH was kept constant at pH = 10 by adding 5 mL of NH_4_OH (35%) at an interval of half an hour. The white slurry obtained (in case of MgAl-LDH) centrifuged at (10,000 rpm, RT, for 10 min) and washed several times with decarbonized water and re-centrifuged to remove any impurities. The obtained gelatinous slurries were re-suspended in 50 mL decarbonated water and placed in a Teflon-stainless reactor to be heated in the oven for 14 h at 80 °C then the products were separated by centrifugation (as before). Finally, the slurries were dried at 80 °C for 48 h to obtain MgAl-LDH and NiAl-LDH powder (Figure 2). In synthesis of Ni LDH, Nickel nitrate hexahydrate Ni (NO_3_)_2_·6H_2_O (0.5 M) was used instead of magnesium nitrate hexahydrates and green slurry obtained as final product of Ni-LDH.

#### 2.1.3. Characterization of LDH

Powder X-ray diffraction (PXRD) patterns of LDH were investigated using X, Pert PRO Panalytical with Cu Kα radiation (λ = 1.5406 Å). Diffraction patterns were at the 2 θ range (4–80) with a scanning rate of 2.4°/min. Particle morphologies of LDH were examined by field transmission electron microscopy (HR-TEM, JSM-7100F); images were recorded with JEOL JEM-2100-115 high-resolution transmission electron microscopes. In each case, the accelerating voltage was 200 kV. The surface images of nanoparticles were then recorded using a Quanta FEG 250 scanning electron microscope (FEI Company, Hillsboro, OR, USA) at EDRC, DRC, Cairo. Samples were mounted onto SEM stubs. The applied SEM conditions were: a 10.1 mm working distance, with an in-lens detector combined with energy-dispersive X-ray spectroscopy (EDX) for the determination of the metal composition. 

#### 2.1.4. Adsorption of Active Ingredient Study

The adsorption experiments of the active ingredient of both *Foeniculum vulgare* (fennel oil) and *Camellia sinensis* (green tea oil) onto MgAl LDH and NiAl LDH were employed at room temperature according to oil miscibility (solubility) using the following protocol, About 2 g of M (II) Al-LDH was dispersed in 50 mL proper solvent, water in case of the adsorption of green tea and chloroform in case of the adsorption of fennel oil, and placed in a three-neck (RBF) followed by degassing through purging nitrogen gas for 10 min. Then, 15 mL of well-mixed (5 mL green tea oil + 10 mL deionized water) in case of green tea oil adsorption which is miscible in water while, well-mixed (5 mL of fennel oil + 10 mL chloroform) in case of fennel oil adsorption which is immiscible in water and miscible in chloroform was added and stirred gently for 8 h. The resulting slurry was centrifuged, washed with a small amount of the same solvent, then well dried at 60 °C for 10 h, grinding with a porcelain mortar was then performed so as to be ready for the fieldwork.

#### 2.1.5. Adsorption Data Analysis and Drug Loading

To investigate the active ingredients, 2 g of LDH loaded essential oil was stirred vigorously for 3 h on absolute ethanol. The slurry collected by centrifugation and supernatant was divided into two portions; one of them concentrated until all the ethanol was vaporized, then a nonpolar solvent was used to dissolve the residue to be analyzed using GC/MS analysis to determine whether any volatile or nonpolar active ingredients were present or not. The other portion was injected directly into the LC MS/MS, to determine the non-volatile or polar active ingredients like polyphenols of flavonoids, using liquid chromatography–electrospray ionization–tandem mass spectrometry (LC-ESI-MS/MS) with an Exion LC AC system for separation and SCIEX Triple Quad 5500 + MS/MS system equipped with an electrospray ionization (ESI) for detection. The separation was performed using ZORBAX SB-C18 Column (4.6 mm × 100 mm, 1.8 µm). The mobile phases consisted of two eluents: A) 0.1% formic acid in water; B) acetonitrile (LC grade). The mobile phase was programmed as following, 2% B from 0–1 min, 2–60% B from 1–21 min, 60% B from 21–25 min, 2% B from 25.01–28 min. The flow rate was 0.8 mL/min and the injection volume was 3 µL. For the MRM analysis of the selected polyphenols, positive and negative ionization modes were applied in the same run with the following parameters: curtain gas: 25 psi; Ion Spray voltage: 4500 and−4500 for positive and negative modes, respectively; source temperature: 400 °C; ion source gas one and two were 55 psi with a declustering potential: 50; collision energy: 25; collision energy spread: 10.

Whereas, the GC/MS analysis was performed by Thermo Scientific, Trace GC Ultra/ISQ Single Quadrupole MS, and TG-5MS fused a silica capillary column (30 m, 0.251 mm, 0.1 mm film thickness). An electron ionization system (ionization energy of 70 eV) was used for the GC/MS detection. Helium, a carrier gas, was used with a constant flow rate of 1 mL/min. The injector and MS transfer line temperature was set at 280 °C. The oven temperature was programmed initially as 50 °C (hold 2 min) to 150 °C at an increasing rate of 7 °C/min, then to 270 at an increasing rate 5 °C/min (hold 2 min), then to 310 °C as a final temperature at an increasing rate of 3.5 °C/min (hold 10 min). The quantification of the identified components was investigated using a percent relative peak area. Tentative identification of the compounds was analyzed by comparing their relative retention time and mass spectra with those of the NIST, WILLY library data of the GC/MS system. Moreover, the identification was accomplished using computer search user-generated reference libraries, incorporating mass spectra. Peaks were examined by single-ion chromatographic reconstruction to confirm their homogeneity. In some cases, when identical spectra have not been found, only the corresponding component’s structural type was proposed based on its mass spectral fragmentation. Reference compounds were co-chromatographed when possible, to confirm GC retention times.

### 2.2. Entomology Work

#### 2.2.1. *Culex pipiens*

*Culex pipiens* larvae were provided from the insectary of the Medical and Molecular Entomology Section, Entomology Department, Faculty of Science, Benha University, Egypt. Mosquito larvae were reared at 27 + 2 °C, 75–85% RH, and 4:10 h (L/D) photoperiod.

#### 2.2.2. In Vitro Larvicidal Efficacy

Crude oils and their nanocomposite were carried out for their larvicidal efficacy [37] against the early fourth instar larvae, *Cx. pipiens.* Oils were added to a solvent consists of dechlorinated water plus 5 mL Tween 20. Twenty larvae were placed in a 500 mL glass beaker containing 250 mL of crude oils or their nanocomposite. Different concentrations of essential oils (125, 250, 500, 1000, and 2000 ppm) were tested [38]. Each experiment and the control group (treated with the solvent only) were replicated three times. Larval mortalities were recorded 0.5, 2, 8, 24, and 48 h post-treatment (PT). 

#### 2.2.3. Larvicidal Field Evaluation

Field evaluation of fennel and green tea oils and their Mg-LDH were evaluated against the larval and pupal mosquito population in stagnant water ditches (avg. 350 m × 4.5 m by 0.65 m deep) at Shablanga village, Qalyubiya Governorate, Egypt, where water was relatively stable with a high mosquito density. The LC_95_ X2 of each oil and its Mg-LDH were applied. The control site was treated with chlorinated water. Three replicates were used for each treatment. Mosquito samples per site were taken prior to treatment and daily PT for a week. The fourth instar larvae in the field water were collected from each site using an enamel plate (450 mL) at each of the larvicides to transport the sample to the laboratory to evaluate the efficacy of the selected larvicides on the mosquito population [39].

#### 2.2.4. In Vitro Adulticidal Efficacy

Adult mosquito susceptibility testing was conducted using modified CDC bottle bioassays [40]. Pure ethanol was used to make different concentrations of each oil (2, 5, 10, 15, and 25%). Oils loaded in Mg-LDH were prepared with different concentrations (0.5, 1, 2, 3, and 4%). The bottles were coated with the desired concentrations and left open to evaporate the solvent overnight at 28 ± 2 °C. Three bottles were used for each concentration. A hand aspirator was used to release adult mosquitoes (10–15, aged 3–4 days) fed on a 10% sucrose solution into each bottle. 10, 20, 30, 40 and 60 min were used as exposure times. The mosquitoes in the bottles were removed. Mosquito groups were placed in separate paper cups containing a 10% sucrose solution, and mortality was measured after 24 h. For each concentration, three replicates were made.

#### 2.2.5. Adulticidal Field Evaluation 

The efficacy and stability of fennel and green tea oils and their Mg-LDH were performed on the adult mosquitoes [41] at Shablanga village, as mentioned before, where animal barns were located inside houses near some irrigation ditches. Each oil and its Mg-LDH (LC_95_ X2) were sprayed in three rooms/home. Three rooms were sprayed with dechlorinated water as a control group. The reduction in adult mosquitoes was calculated [42]. To determine the persistence or stability of selected adulticidal, ten adult mosquitoes from the same house were captured through the aspirator and put into a device on the wall in the room for 30 min. Then, the last adult mosquito was killed (WHO 2016).

#### 2.2.6. The Efficacy against Non-Target Predators

An evaluation of the larvicidal and adulticidal activity of fennel and green tea oil was conducted, before and after being loaded by layered double hydroxide (LDH) in both laboratory and field conditions. The efficiency of fennel and green tea oils were examined against some selected common predators captured using a standing diver in different mosquito larval habitats as *Gambosia affinis*, *Cybister tripunctatus,* and *Sphaerodema urinator*. The predators were collected alive in plastic bags half-filled with water from the field to the laboratory and identified in the Entomology Department at Benha University, Egypt [43].

#### 2.2.7. Data Analysis

For analyzing the data, we applied the one-way analysis of variance (ANOVA), and multiple comparisons were carried out applying Tukey’s test and Probit analysis for calculating the lethal values using the computer program PASW Statistics 2009 (SPSS version 22). The significance level was set at *p* < 0.05.

The percent reduction in larval and pupal density was calculated [42] according to the following formula:

% reduction = 100 − {(C1 × T2)/(C2 × T1)} × 100

where C1 = Pre-treatment immature density in the control site.

C2 = post-treatment immature density in the control site.

T1 = pre-treatment immature density in the treated site.

T2 = post-treatment immature density in the treated site.

## 3. Results

### 3.1. Synthesis of M(II)LDH

#### 3.1.1. Characterization of M(II)LDH

**Figure 3:**
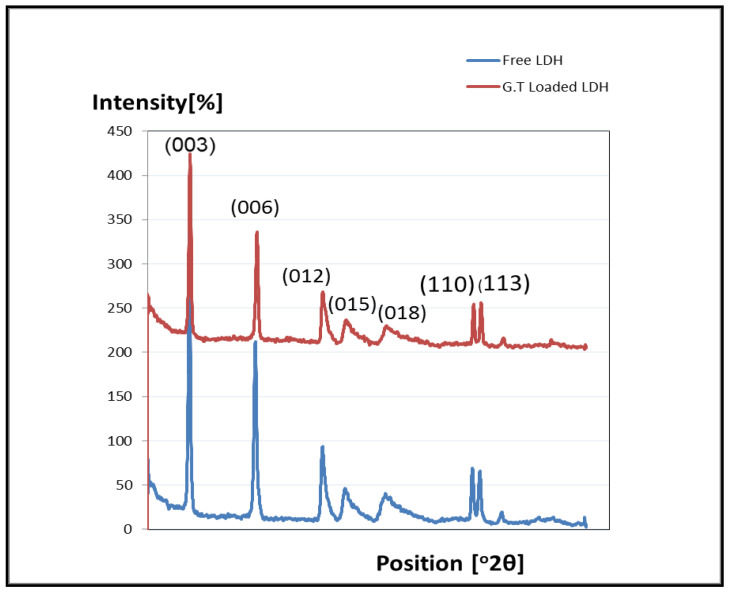
PXRD Diffraction pattern of unloaded MgAl-LDH and loaded green tea oil.

The phase determination of synthesized LDH hydrotalcite- like materials was conducted by using XRD. Figure 3 shows the powdered X-ray Diffraction Patterns of unloaded MgAl-LDH and loaded MgAl-LDH with green tea oil. The diffraction pattern confirmed that, LDH synthesized perfectly with high crystallinity due to the appearance of strong and sharp peaks in both Mg LDH and MgLDH-GT. The values of 2θ = 11, 23, 34, 38, 45 and 60 were attributable to (003), (006), (009), (015), (012), (110) and (113) planes of reflection specific to MgAl-LDH. P-XRD diffractogram of the synthesized MgAl LDH revealed a hydrotalcite structure with characteristic reflections, starting from intense and sharp basal 00l reflections of 003, 006 and 009 planes that showed a low reflection angle, less than 35 (35° > 2θ). Whereas, little broad 0kl planes of reflection of 012 and 015 clearly showed moderate reflection angle in range of (2θ = 38° and 45°), and finally, the sharp hk0 and hkl planes of reflections of 110 and 113, in the highest region angle (2θ = 45–60°). Referring to the XRD library, the data obtained fund to be in consistent with the structure of Mg Al LDH.

**Figure 4:**
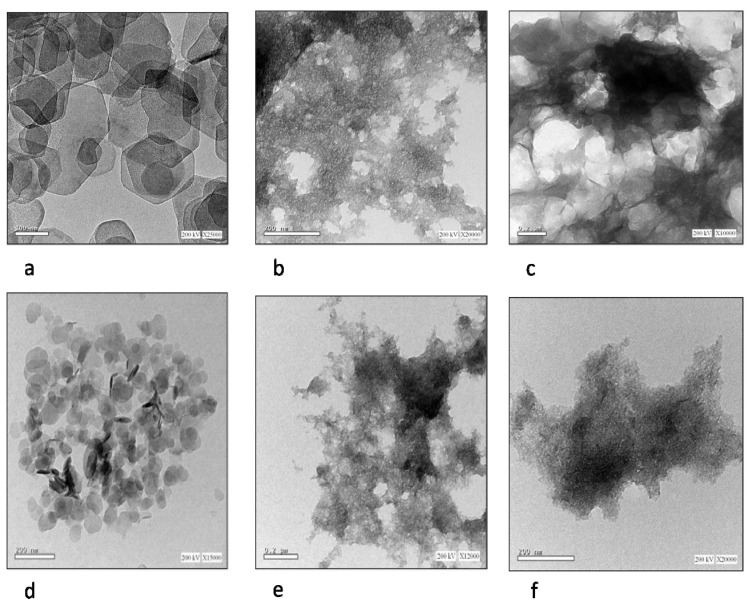
(**a**) represents TEM of synthesized MgAl-LDH (MgLDH), (**b**) represents TEM of synthesized NiAl-LDH (NiLDH), (**c**) represents TEM of synthesized MgAl-LDH intercalated green tea oil (MgLDH-GT), (**d**) represents TEM of synthesized MgAl-LDH intercalated fennel oil (MgLDH-F), (**e**) represents TEM of synthesized NiAl-LDH intercalated green tea oil (NiLDH-GT), (**f**) represents TEM of synthesized NiAl-LDH intercalated fennel oil (NiLDH-F).

**Figure 5:**
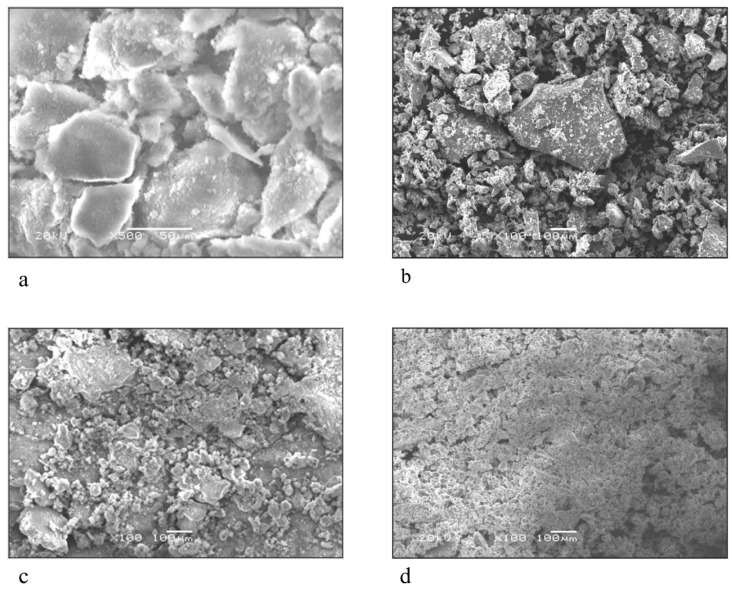
(**a**) represents SEM of synthesized MgAl-LDH (MgLDH), (**b**) represents SEM of synthesized NiAl-LDH (NiLDH), (**c**) represents SEM of synthesized MgAl-LDH intercalated green tea oil (MgLDH-GT), (**d**) TEM of synthesized NiAl-LDH intercalated green tea (NiLDH-GT).

**Figure 6:**
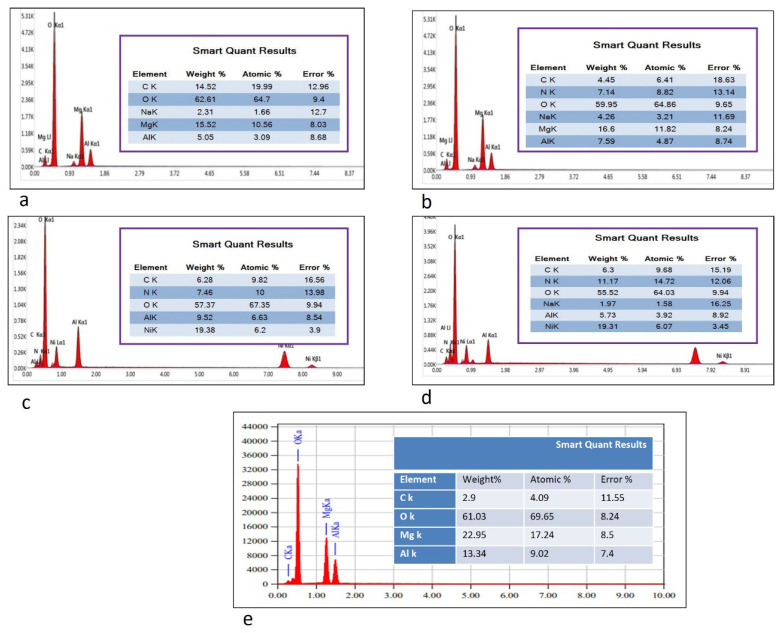
(**a**) represents SEM-EDX component of synthesized MgLDH-GT, (**b**) represents SEM-EDX the elemental structur of synthesized MgLDH-F, (**c**) represents SEM-EDX the elemental structure of synthesized NiLDH-GT, (**d**) represents SEM-EDX the elemental structure of synthesized NiLDH-F, (**e**) represents SEM-EDX the elemental structur of synthesized MgAl-LDH itself (unloaded).

**Figure 7:**
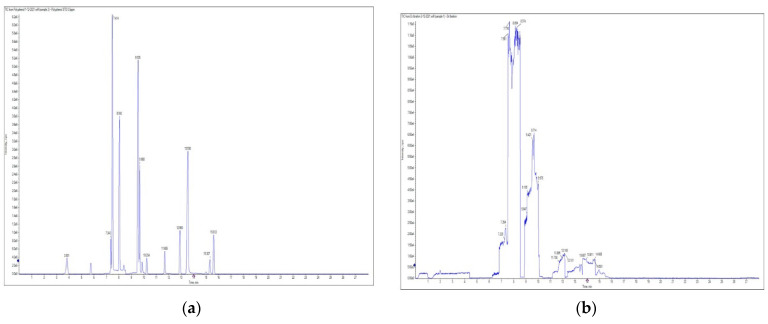
(**a**) represents LC MS/MS of standards and (**b**) represents LC MS/MS of synthesized MgAl-LDH intercalated Green tea oil (MgLDH-GT) after desorption using ethanol.

The morphological and microstructural of LDHs investigated Using Transmission Electron Microscopy (TEM) showed, as in Figure 4, a well-designed hexagonal shape with a particle size dimension of 100 nm for MgAl-LDH (Figure 4a), and a thin plate structure for NiAl-LDH with a particle size 200 nm (Figure 4b), respectively. Mg and Ni LDH intercalated green tea and fennel oil showed the bulky aggregations on a TEM micrograph, as showed in Figure 4c–f, due to the high absorbability made by LDH with little increase in particle size, especially in the case of MgAl-LDH intercalated green tea as, 0.2 µm, 200 nm, 0.2 µm and 200 nm for MgLDH-GT, MgLDH-F, NiLDH-GT and NiLDH-F, respectively. To investigate the surface morphology, field-emission scanning electron microscopy (FESEM) was used. Figure 5 revealed that the unloaded MgLDH and NiLDH appeared in two types: sphere-like and plate-like in the order of 50 µm to 100 µm, respectively. In addition, SEM-EDX was used to determine the elemental analysis of the metal surface constituents or atomic composition for the MgLDH and NiLDH intercalated green tea and fennel oils as shown in (Figure 6). Based on, the SEM-EDX results, the best loading occurred between MgAl-LDH and green tea oil, which may explain the potential larvicidal and adulticidal actions of MgLDH-GT against *Cx. pipiens* and the related non-target species. That interpretation comes from the weight percentage of the carbon atom present on the surface of the sample at a point as the higher the carbon content, the higher the active ingredients loaded. The loaded MgLDH-GT presented a carbon percentage of 14.52% while unloaded MgLDH showed 2.9% (Figure 6a,e), whereas 4.45, 6.28 and 6.3% were recorded for the carbon content of MgLDH-F, NiLDH-GT and NiLDH-F, respectively, as shown in (Figure 6b–d). The LCMS/MS results, shown in Figure 7, are consistent with the SEM-EDX results shown in Figure 6, that means when the number of active ingredients detected by LCMS/MS increased the carbon weight percentage detected by SEM-EDX also increased. That will be observed if we compare SEM-EDX of unloaded Mg LDH, which contains a low carbon content of 2.9%, and Mg LDH loaded green tea oil that contains a high carbon content of 14.52%. The higher carbon content explains the more active ingredient loading.

#### 3.1.2. Desorption of Active Ingredient and Drug Loading

To ensure the existence of volatile and nonvolatile active ingredients released from nanoclay systems and to rationalize the insecticidal activity reasons, the desorption was conducted as explained in the experimental section using ethanol as the solvent and Mg LDH loaded green tea as template of nanoclays loaded with natural oil followed by an analysis of the supernatant produced from the desorption process using a chromatography technique. Results of volatile active ingredients determined by GC/MS showed neglected very small uproarious signals (background) in a chromatogram, confirming that there are no significant peaks related to volatile active ingredients. This may be due to the low affinity of LDHs nanoclays towards volatile active ingredients or the reason for the absence of this volatile compound may be they not having the opportunity to be loaded as a result of their high volatility.

Polyphenol and flavonoid nonvolatile active ingredients were detected using LC MS/MS. According to Figure 7 and Table 1, significant peaks related to polyphenols and flavonoids of Vanillin, Catechin, Daidzein, Ellagic acid, Naringenin, Myricetin and Syringic acid with concentrations of 0.76, 0.73, 0.67, 0.59, 0.52, 0.44 and 0.42 μg/g, respectively, were shown compared to their standard concentration of 0.05 g/mL (Figure 7a,b). The detected flavonoids and polyphenols, as active ingredients, were proven to have insecticidal effect in many oil and plant extracts [44,45]. The last findings explain the potential insecticidal activities of MgLDH loaded green tea oil. These findings are consistent with those announced in our previous study [46], which discussed the results of the HPLC analysis of raw green tea oil (*Camellia sinensis*) and confirmed that green tea oil contains some polyphenolic and flavonoid active ingredients, which transferred to the MgLDH nanoclays and were effectively released to be detected using LCMS/MS in our current study.

### 3.2. Larval Laboratory Experiments

The larvicidal effects of fennel, *F. vulgare* and green tea oils, *C. sinensis* and their LDH were evaluated against the early 4th larvae, *Cx. pipiens*. The mortality (MO) % PT with 2000 ppm for 48 h with fennel oil, and its LDH reached 100% MO (Table 2), whereas MO% for green tea oil, Mg-LDH-GT, and Ni-LDH-GT were 95.00, 100.00, and 96.67%, respectively (Table 3).

The LC_50_ and LC_95_ values were calculated for fennel oil (843.88 and 1989.29 ppm), Mg-LDH-F (451.95 and 1001.75 ppm) and Ni-LDH-F (550.12 and 1276.82 ppm), and green tea oil (938.93 and 2100.75 ppm), Mg-LDH-GT (530.46 and 1301.02 ppm), and Ni-LDH-GT (769.94 and 1878.79 ppm) (Table 4). The data LC_50_ values of fennel and green tea nanoparticles (Mg and Ni LDH) and fennel and green tea oils showed there are significant differences (F = 13.4; df = 2,27; *p* = 0.001) between all treatments except the control.

### 3.3. Larvicidal Field Evaluation

**Figure 8:**
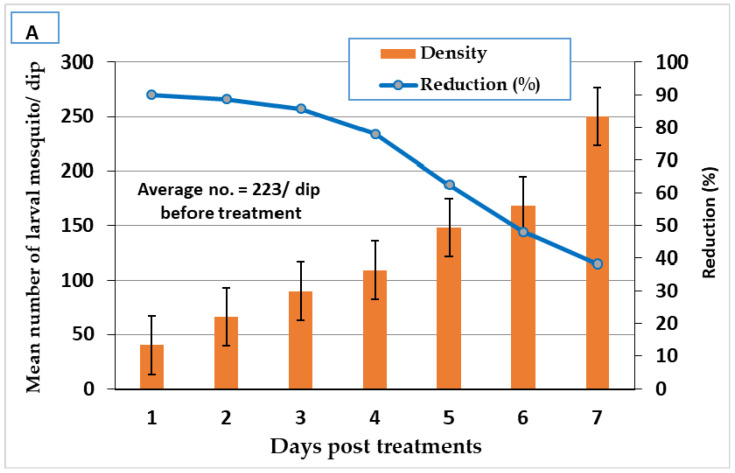

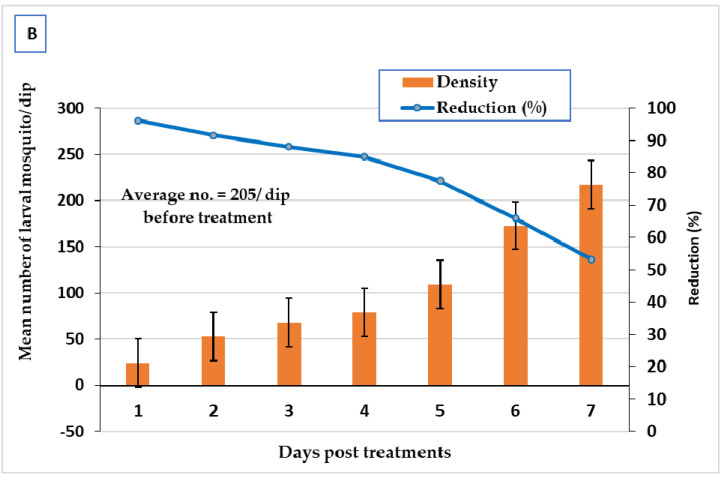
Field efficacy of fennel oil (**A**) and its Mg-LDH (**B**) treated at dose of LC_95_ X2 (3978.6 and 2003.5 ppm, respectively, in larval breeding sites.

**Figure 9:**
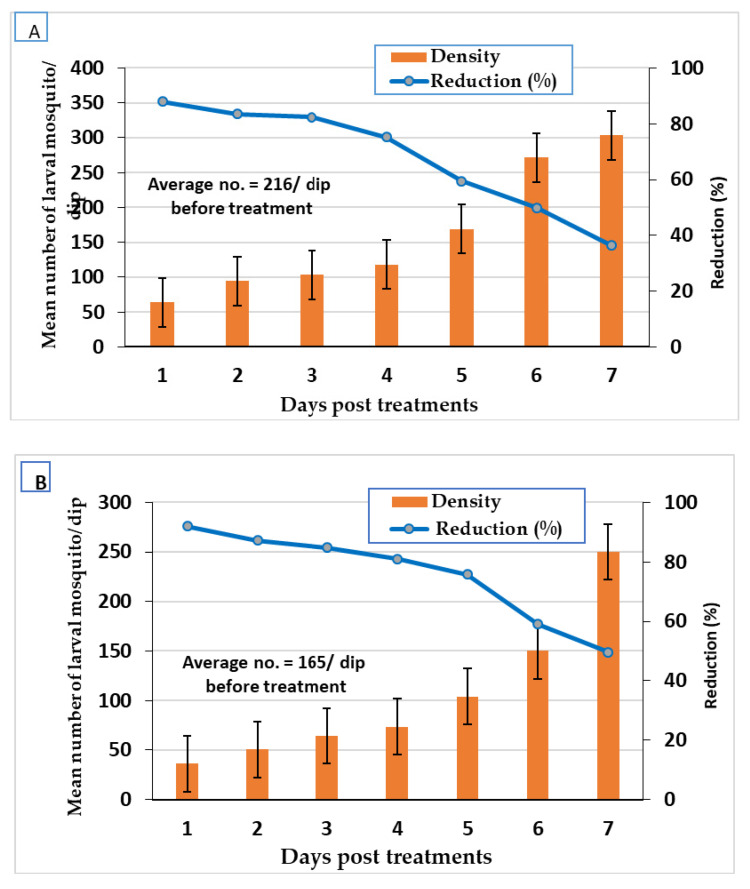
Field efficacy of green tea oil (**A**) and its Mg-LDH (**B**) treated at dose of LC_95_ X2 (4201.5 and 2602.04 ppm, respectively) in larval breeding sites.

Fennel and green tea oils and their Mg-LDH lowered larval density in small ditches at Shablanga village PT with doses of LC_95_ X2 for fennel oil and Mg-LDH fennel (3978.6 and 2003.5 ppm, respectively), and for green tea oil and Mg-LDH-GT (4201.5 and 2602.04 ppm, respectively). Treatments reduced larvae density, where the larval reduction % of fennel oil and Mg-LDH-F reached 90.1 and 96.2%, 24 h PT and their persistence (>50%) reached 5 and 7 days PT, respectively (Figure 8). The reduction percentage of green tea oil and Mg-LDH-GT reached 88.00 and 90.01%, 24 h PT and their persistence (reduction% > 50%) reached 5 and 6 days PT, respectively (Figure 9). 

### 3.4. In Vitro Adulticidal Effect 

The adulticidal effects of the applied materials was evaluated against the *Cx. pipiens* (3–4 days old) and indicated that Mg-LDH-F and Ni-LDH-F were more highly effective than fennel oil, where the mortality reached 100%, 93.3% and 86.7%, 24 h PT, respectively, and their LC_95_ values were 14.16, 33.16, 72.40%, respectively (Table 5). 

Moreover, Mg-LDH-GT and Ni-LDH-GT were more effective than green tea oil, where the mortality reached 93.3%, 83.33%, and 76.67%, 24 h PT, respectively, and their LC_95_ values were 8.84, 29.14, and 35.39%, respectively (Table 6).

### 3.5. Adult Field Experiments

**Figure 10:**
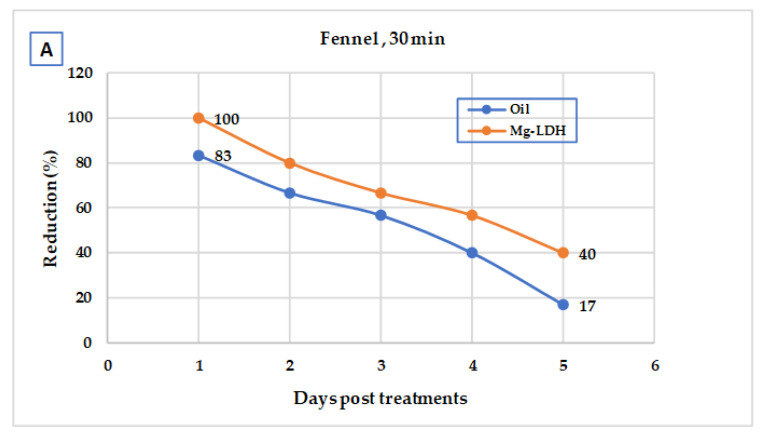

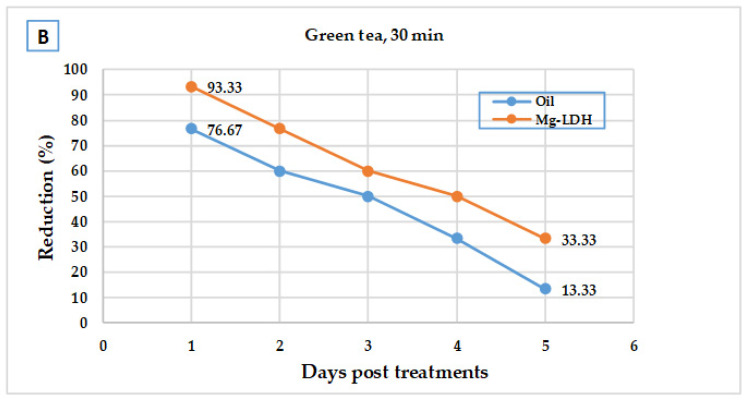
Persistence of Fennel oil and its Mg-LDH fennel (**A**), and green tea oil and its Mg-LDH tea (**B**) against the adult mosquito in treated homes, 30 min post exposure for 5 days.

After spraying with LC_95_ X2 for 15 min, the reduction in adult density PT with fennel oil, Mg-LDH-F, green tea oil, and Mg-LDH-GT were 83.1, 100, 77, 99.0%, respectively, with persistence (>50%) lasting for three days (Table 7, Figure 10).

### 3.6. The Efficacy against Non-Target Predators

The efficiency of oils and their Mg-LDH were examined against some predators, such as *G. affinis, C. tripunctatus,* and *S. urinator.* There was no significant difference between the mean of predation of both fennel and green tea oils. In the case of Mg-LDH-GT and Mg-LDH-F, the predation rate of the beetle, *C. tripunctatus* increased (71% and 69%), respectively, compared to green tea and fennel oils (55.67, and 52.33, respectively). On the other hand, the predation rate without treatments reached >97% (Table 8).

## 4. Discussion

The most efficient strategy to prevent mosquito bites and disease transmission is synthetic insecticides to kill larvae in water bodies and adults in the air. To avoid pest resistance and environmental contamination, natural alternatives are in high demand. EOs and plant extracts are relatively safe, readily available, and biodegradable [26] and could be used as natural alternatives for mosquito control [47,48,49].

Our results indicated that the fennel oil, *F. vulgare* and green tea oil, and *C. sinensis* oils have larvicidal and adulticidal activity, and their LDH Nanoclays increased their efficacy and persistence.

Our data revealed that fennel oil effectively controlled larvae and adults of *Cx. pipiens* (LC_50_ = 843.88 ppm and 3.25%, respectively). The novel fennel was more effective than the oil form against larvae and adults, represented by Mg-LDH-F (LC_50_ = 451.95 ppm and 1.75%, respectively) and Ni- LDH-F (LC_50_ = 550.12 ppm and 2.30%, respectively), (F = 13.4; df = 2,27; *p* = 0.001).

Similar to our findings, fennel oil was effective against larvae and adults of *Culex quinquefasciatus* [36,50], *Anopheles atroparvus* [51], and *Aedes aegypti* [35]. In another study, Zoubiri, et al. [52] showed that 40 mg/L of fennel oil, *F. vulgare* oil, was sufficient to cause 50% mortality in the second larval instar of *Cx. pipiens* after 2 h of exposure. Furthermore, a concentration of 60 mg/L resulted in 90% mortality for the fourth instar larvae after 4 h of exposure.

The present study revealed that green tea oil effectively controlled larvae and adults of *Cx. pipiens* (LC_50_ = 938.93 ppm and 5.45%, respectively). The novel green tea LDHs were more effective than the crude oil against larvae and adults represented by Mg- LDH-GT (LC_50_ = 530.46 ppm and 0.42%, respectively) and Ni- LDH-GT (LC_50_ = 769.94 ppm and 0.45%, respectively). Similarly, the leaf extract of green tea showed a larvicidal effect against *Anopheles arabiensis* and *Anopheles gambiae* (*s.s*.) [33] and induced larvicidal (100% larval mortality at 1000 ppm) and adult repellent effects against *Cx. pipiens,* providing 100% protection from the female bites at 6 mg/cm^2^ [53]. Moreover, green tea is an effective larvicide against *Drosophila prosaltans* due to caffeine and other organic compounds in its water extracts [54].

Some other oils and plant extracts induced a similar effect against *Cx. pipiens* larvae. *Nigella sativa*, *Allium cepa,* and *Sesamum indicum* showed larvicidal results against *Cx. pipiens* laboratory and field strains in Egypt (LC_50_ values were 247.99 and 108.63; 32.11 and 2.87; and 673.22 and 143.87 ppm, respectively); such oils also affected pupation and adult emergence rates and induced developmental malformations [55]. Oils effectively controlled *Cx. pipiens* larvae as fenugreek (*Trigonella foenum-grecum*), earth almond (*Cyperus esculentus*), mustard (*Brassica compestris*), olibanum (*Boswellia serrata*), rocket (*Eruca sativa*), and parsley (*Carum ptroselinum*) (LC_50_ = 32.42, 47.17, 71.37, and 83.36, 86.06, and 152.94 ppm, respectively), and suppressed pupation and adult emergence rates [56]. Larvicidal effects against *Cx. pipiens* expressed by Oil-resins as *Araucaria heterophylla, Commiphora molmol, Eucalyptus camaldulensis, Pistacia lentiscus,* and *Boswellia sacra* [49].

A similar adulticidal effect was recorded via topical application of EOs against laboratory and natural field strains of *Ae. Aegypti*, indicating that the laboratory strain was slightly more susceptible to EOs than the natural field strain with no statistical difference. The highest effect was induced by caraway, followed by zedoary, celery, long pepper, and Chinese star anise (LC_50_ = 5.44, 5.94, 5.96, 6.21, and 8.52 μg/mg female, respectively, for the laboratory strain and 5.54, 6.02, 6.14, 6.35, and 8.83 μg/mg female, respectively, for the field strain [57].

Some oils act as adult mosquito repellents; Zanthoxylum *piperitum* oil alone and oil plus 5% vanillin repelled laboratory-reared female *Ae. aegypti* (median protection times = 1.5 and 2.5 h, respectively). Under field conditions, Z. *piperitum* oil + 5% vanillin was found to provide better protection against *Aedes gardnerii*, *Anopheles barbirostris*, *Armigeres subalbatus*, *Cx. tritaeniorhynchus*, *Cx. gelidus*, *Cx. vishnui* group, and *Mansonia uniformis)* than 25% DEET + 5% vanillin [58].

It has been reviewed that most one-pot plant-fabricated polydisperse metal nanoparticles were highly effective as mosquito larvicides, pupicides, and adulticides with very low LC_50_ values (1–30 mg/L) [59]. Similar findings were recorded for different AgNPs against mosquito larvae as *Artemisia vulgaris* AgNP against larvae and pupae of *Ae. aegypti* [60]; *Hypnea musciformis* AgNPs against *Ae. aegypti* [47]; *Nicandra physalodes* Ag NPs against *Anopheles stephensi*, *Ae. aegypti* and *C. quinquefasciatus* [61]; *Zornia diphylla* AgNP against *Anopheles subpictus, Aedes albopictus,* and *Culex tritaeniorhynchus* [62]; *Cyprus rotundas NgNPs against Ae. albopictus*, *An. stephensi* and *C. quinquefasciatus* [63].

Moreover, larval, pupal periods, and adult longevity were adversely affected PT with crude extracts and AgNP of *Azadirachta indica, Datura stramonium, Zingiber officinale, Syzygium aromaticum* and their synthesized AgNPs against *Cx. pipiens* [64]. Reduced adult emergence rates of *Aedes aegypti* and *C. quinquefasciatus* were recorded PT with the aqueous leaf extract of *Adiantum raddianum* and its green synthesized AgNPs [65] such as silver, protein-lipid, nanoparticles (Ag-PL NPs) (core-shell), fabricated from the seed extract from an almond tree, *Sterculia foetida* (LC_50_ < 4.5 ppm) against larvae of *Ae. aegypti*, *Anopheles stephensi*, and *C. quinquefasciatus* [66].

With almost half of the doses applied in the field, the larval reduction percentage of fennel oil and Mg-LDH-F reduced larval density (92% and 96.2%, 24 h PT) and their effect lasted (reduction% > 50%) for 5 and 7 days PT, respectively. Those of green tea oil and Mg-LDH-GT were 88.00 and 90.01%, respectively, 24 h PT and persisted for six days PT, respectively. On the other hand, the reduction in adult density PT with fennel oil, Mg-LDH-F, green tea oil, and Mg-LDH-GT reached 83.00, 100, 76.67, and 97.00%, respectively, and was effective for three days. To the best of our knowledge, there was no previously filed application for the use of nanoparticles against mosquitoes.

The tea leaves *C. sinensis* are used to produce a non-alcoholic drink that really is drunk all over the world for its psycho-activity and health benefits [33]. Graham (1992) found that the immature tea leaves are high in methylxanthines (caffeine, theophylline), catechins (catechin, gallocatechin, catechin gallate), flavonoids, vitamins, proteins, glycosides (kaempferol, myricetin), and that the catechins have antimalarial, antiviral, antibacterial, anticarcinogenic, antioxidant, anti-inflammatory, anti-arthritis, anti-aging properties (Sannella et al., 2007; Afzal et al., 2015). Proanthocyanidins were found to be the most abundant bioactive chemicals in the *C. sinensis* leaf extract [33].

Plant compounds (flavonoids, alkaloids, esters, glycosides, and fatty acids) have anti-insect effects on the chemical compounds used in the elimination of insects in various ways, as discussed by Baz et al., [67], including feeding deterrents/antifeedants, toxicants, growth retardants, repellents, chemosterilants, and attractants [26,27,68].

Mosquito predators, such as *Gambusia* sp., are released worldwide for biological mosquito control due to their excellent predation effectiveness [59]. This work indicated the safety of the applied materials against three non-target organisms. Similarly, several studies have investigated the impact of the acute toxicity of nanoparticles on the aquatic non-target species, and did not detect toxicity within the silver nanoparticles produced using plant extracts toxic to mosquito larvae [69].

The present work indicated that the predation rates of *C. tripunctatus* and *S. urinator* were slightly decreased and increased after being subjected to crude oils and LDH, respectively, when compared to the free Mg-LDH control group. Correspondingly, a noticed increase in the predation rate of the tadpoles, *Hoplobatrachus tigerinus*, against the larvae of *Ae. aegypti*, in the laboratory and in an aquatic environment, treated with ultra-low doses of AgNP, was recorded [60]. AgNP of *Nicandra physalodes* was safe for the non-target aquatic organism *Diplonychus indicus* (LC_50_ and LC_90_ values were 1032.81 and 19,076.59 μg/mL, respectively) [61]. *Zornia diphylla* AgNP is safe for some non-target organisms as *Chironomus circumdatus, Anisops bouvieri* and *Gambusia affinis* (LC_50_ = 613.11 − 6903.93 μg/mL), if compared to target mosquitoes [62].

Some oils have a neurotoxic effect and interfere with the neuromodulator octopamine and gamma-aminobutyric acid, GABA, and gated chloride channels [27]. Few studies reveal the mode of action of green-synthesized nanoparticles, which may be related to exoskeleton penetration, or nanoparticles that could bind to sulfur from proteins or to phosphorus from DNA, inducing the rapid denaturation of enzymes and organelles, followed by the decrease in membrane permeability and disturbance in proton motive force affecting cellular function and cell death [59]. In addition, nanoparticles enter the cuticle membrane of mosquito larvae, then move to their intestine and damage their DNA banding pattern [63].

Fennel oil has Estragole (70.36%) and Limonene (8.96%) (Our unpublished work). Limonene is a cyclic monoterpene with insecticidal effect [70] and Estragole is toxic to adult fruit flies, *Ceratitis capitata* [71].

## Figures and Tables

**Table 1:** LC MS/MS adsorbent composition of the active ingredient from MgAl-LDH intercalated (Green Tea oil) after desorption by ethanol compared to standards.

Compounds	Retention Time	Std Conc. (μg/mL)	Samples (Conc. μg/g)	Structure
Chlorogenic acid	7.30	0.05	0.23	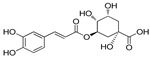
Daidzein	12.81	0.05	0.67	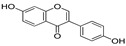
Gallic acid	Not Detected	0.05	Not Detected	-
Caffeic acid	Not Detected	0.05	Not Detected	-
Rutin	Not Detected	0.05	Not Detected	-
Coumaric acid	Not Detected	0.05	Not Detected	-
Vanillin	9.47	0.05	0.76	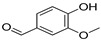
Naringenin	14.89	0.05	0.52	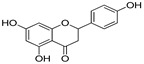
Querectin	Not Detected	0.05	Not Detected	-
Ellagic acid	9.86	0.05	0.59	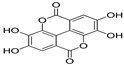
3,4-Dihydroxybenzoic acid	Not Detected	0.05	Not Detected	-
Hesperetin	Not Detected	0.05	Not Detected	-
Myricetin	11.62	0.05	0.44	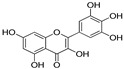
Cinnamic acid	Not Detected	0.05	Not Detected	-
Methyl gallate	Not Detected	0.05	Not Detected	-
Kaempferol	Not Detected	0.05	Not Detected	-
Ferulic acid	Not Detected	0.05	Not Detected	-
Syringic acid	8.35	0.05	0.42	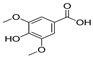
Apigenin	Not Detected	0.05	Not Detected	-
Catechin	7.30	0.05	0.73	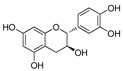
Luteolin	Not Detected	0.05	Not Detected	-

**Table 2:** Larvicidal effects of fennel oil and its LDH against *Culex pipiens*, 48 h post treatments.

Oil Name	Nanocomposite	Time (Hour)	Mortality % (Mean ± SE)				
			125 *	250 *	500 *	1000 *	2000 *
Fennel (*Foeniculum vulgare*)	Oil	0.5	0.00 ± 0.00 ^c^	0.00 ± 0.00 ^c^	3.33 ± 0.66 ^d^	8.33 ± 1.66 ^e^	13.33 ± 0.33 ^e^
		2	1.67 ± 0.33 ^c^	3.33 ± 0.33d ^c^	6.67 ± 0.33 ^d^	21.67 ± 0.33 ^d^	30.0 ± 0.57 ^d^
		8	3.33 ± 0.33 ^c^	8.33 ± 0.33 ^c^	18.33 ± 0.33 ^c^	45.0 ± 1.15 ^c^	61.66 ± 1.20 ^c^
		24	8.33 ± 0.33 ^b^	20.0 ± 0.57 ^b^	60.0 ± 0.57 ^b^	60.0 ± 0.57 ^b^	90.0 ± 0.00 ^a^
		48	13.33 ± 0.33 ^a^	33.33 ± 0.33 ^a^	61.33 ± 0.66 ^a^	86.67 ± 0.66 ^a^	100.0 ± 0.00 ^a^
	Mg-LDH-F	0.5	3.33 ± 0.33 ^c^	10.0 ± 0.00 ^c^	13.33 ± 0.33 ^d^	25.0 ± 0.57 ^d^	31.67 ± 0.33 ^d^
		2	6.67 ± 0.33 ^bc^	13.33 ± 0.33 ^c^	20.0 ± 0.58 ^d^	40.0 ± 0.57 ^c^	50.0 ± 0.57 ^c^
		8	10.0 ± 0.57 ^b^	26.67 ± 0.88 ^b^	51.67 ± 0.67 ^c^	70.0 ± 0.57 ^b^	83.33 ± 0.33 ^b^
		24	16.67 ± 0.33 ^a^	35.0 ± 0.57 ^ab^	65.0 ± 0.57 ^b^	90.0 ± 0.57 ^a^	100.0 ± 0.0 ^a^
		48	18.33 ± 0.33 ^a^	40.0 ± 0.57 ^a^	75.0 ± 0.57 ^a^	96.67 ± 0.33 ^a^	100.0 ± 0.00 ^a^
	Ni-LDH-F	0.5	1.67 ± 0.33 ^b^	5.0 ± 0.00 ^c^	11.67 ± 0.33 ^c^	16.67 ± 0.33 ^d^	20.0 ± 0.57 ^d^
		2	5.0 ± 0.00 ^b^	8.33 ± 0.33 ^c^	15.0 ± 0.58 ^c^	33.33 ± 0.88 ^c^	40.0 ± 1.0 ^c^
		8	8.33 ± 0.67 ^ab^	21.67 ± 0.33 ^b^	46.67 ± 0.88 ^b^	65.0 ± 0.57 ^b^	75.0 ± 0.57 ^a^
		24	13.33 ± 0.33 ^a^	30.0 ± 0.57 ^a^	60.0 ± 0.57 ^a^	83.33 ± 0.33 ^a^	98.33 ± 0.33 ^a^
		48	15.0 ± 0.57 ^a^	35.0 ± 0.57 ^a^	66.67 ± 0.88 ^a^	90.0 ± 0.57 ^a^	100.0 ± 0.00 ^a^
	Mg-Free	0.5	0.00 ± 0.00 ^c^	0.00 ± 0.00 ^c^	1.67 ± 0.33 ^d^	3.33 ± 0.33 ^e^	5.0 ± 0.00 ^f^
		2	0.00 ± 0.00 ^c^	1.67 ± 0.33 ^c^	1.67 ± 0.33 ^d^	3.33 ± 0.33 ^e^	8.33 ± 0.33 ^f^
		8	0.00 ± 0.00 ^c^	1.67 ± 0.33 ^c^	3.33 ± 0.33 ^d^	5.0 ± 0.00 ^e^	8.33 ± 0.33 ^f^
		24	0.00 ± 0.00 ^c^	1.67 ± 0.33 ^c^	3.33 ± 0.33 ^d^	5.0 ± 0.00 ^e^	8.33 ± 0.33 ^f^
		48	0.00 ± 0.00 ^c^	1.67 ± 0.33 ^c^	3.33 ± 0.33 ^d^	5.0 ± 0.00 ^e^	10.0 ± 0.00 ^e^

a, b and c… etc.: There is no significant difference (*p* > 0.05) between any two means, within the same column that have the same superscript letter (one-way ANOVA, Tukey’s range test, *p* > 0.05). * Conc. (ppm).

**Table 3:** Larvicidal effects of green tea oil and its LDH against *Culex pipiens*, 48 h post treatments.

Oil Name	Nanocomposite	Time (Hour)	Mortality % (Mean ± SE)				
			125 *	250 *	500 *	1000 *	2000 *
Green tea (*Camellia sinensis*)	Oil	0.5	0.00 ± 0.00 ^c^	1.67 ± 0.33 ^c^	3.33 ± 0.33 ^d^	6.67 ± 0.33 ^e^	11.66 ± 0.33 ^e^
		2	1.67 ± 0.33 ^bc^	3.33 ± 0.33 ^c^	5.0 ± 0.00 ^d^	18.33 ± 0.33 ^d^	28.33 ± 0.66 ^d^
		8	3.33 ± 0.33 ^bc^	8.33 ± 0.33 ^c^	15.0 ± 0.00 ^c^	41.67 ± 0.88 ^c^	53.33 ± 0.88 ^c^
		24	5.0 ± 0.00 ^b^	16.67 ± 0.33 ^b^	28.33 ± 0.33 ^b^	56.67 ± 0.88 ^b^	85.0 ± 0.57 ^b^
		48	13.33 ± 0.33 ^a^	31.67 ± 0.66 ^a^	53.33 ± 0.33 ^a^	86.6 ± 0.88 ^a^	95.00 ± 0.57^a^
	Mg-LDH-GT	0.5	3.33 ± 0.33 ^d^	8.33 ± 0.33 ^c^	11.67 ± 0.33 ^c^	21.67 ± 0.33 ^d^	28.33 ± 0.88 ^d^
		2	6.67 ± 0.33 ^bc^	10.0 ± 0.57 ^c^	16.67 ± 0.33 ^c^	36.67 ± 0.57 ^c^	46.67 ± 0.33 ^c^
		8	11.67 ± 0.33 ^bc^	26.67 ± 0.33 ^b^	45.0 ± 0.57 ^b^	68.33 ± 0.88 ^b^	81.67 ± 0.88 ^b^
		24	18.33 ± 0.67 ^ab^	36.67 ± 0.33 ^a^	58.33 ± 0.88 ^a^	83.33 ± 0.67 ^a^	98.33 ± 0.33 ^a^
		48	20.0 ± 0.57 ^a^	41.67 ± 0.33 ^a^	66.67 ± 0.33 ^a^	88.33 ± 0.33 ^a^	100.0 ± 0.00 ^a^
	Ni-LDH-GT	0.5	1.67 ± 0.33 ^c^	3.33 ± 0.33 ^d^	8.33 ± 0.33 ^c^	11.67 ± 0.33 ^d^	16.67 ± 0.67 ^d^
		2	3.33 ± 0.33 ^bc^	8.33 ± 0.33 ^cd^	10.0 ± 0.58 ^c^	25.0 ± 0.58 ^c^	35.0 ± 0.57 ^c^
		8	6.67 ± 0.33 ^ab^	13.33 ± 0.33 ^bc^	28.33 ± 0.33 ^b^	48.33 ± 0.33 ^b^	65.0 ± 0.57 ^b^
		24	8.33 ± 0.33 ^a^	18.33 ± 0.33 ^b^	40.0 ± 0.57 ^a^	63.33 ± 0.57 ^a^	93.33 ± 0.33 ^a^
		48	10.0 ± 0.00 ^a^	21.67 ± 0.57 ^a^	43.33 ± 0.33 ^a^	75.00 ± 0.00 ^a^	96.67 ± 0.33 ^a^
	Ni-Free	0.5	0.00 ± 0.00 ^c^	0.00 ± 0.00 ^c^	1.67 ± 0.33 ^d^	3.33 ± 0.33 ^e^	5.0 ± 0.00 ^f^
		2	0.00 ± 0.00 ^c^	00.00 ± 0.00 ^c^	1.67 ± 0.33 ^d^	3.33 ± 0.33 ^e^	5.0 ± 0.00 ^f^
		8	0.00 ± 0.00 ^c^	1.67 ± 0.33 ^c^	3.33 ± 0.33 ^d^	3.33 ± 0.33 ^e^	8.33 ± 0.33 ^f^
		24	0.00 ± 0.00 ^c^	1.67 ± 0.33 ^c^	3.33 ± 0.33 ^d^	5.0 ± 0.00 ^e^	8.33 ± 0.33 ^f^
		48	0.00 ± 0.00 ^c^	1.67 ± 0.33 ^c^	3.33 ± 0.33 ^d^	5.0 ± 0.00 ^e^	8.33 ± 0.33 ^f^

a, b and c… etc.: There is no significant difference (*p* > 0.05) between any two means, within the same column that have the same superscript letter (one-way ANOVA, Tukey’s range test, *p* > 0.05). * Conc. (ppm).

**Table 4:** The larvicidal effects of fennel, green tea oils and its LDH formulations against *Culex pipiens*, 24 h post treatments.

Oil Name	Nanocomposite	LC_50_ (Low.–Up.) *	LC_90_ (Low.–Up.)	LC_95_ (Low.–Up.)	Chi (Sig)	Equation
Fennel (*Foeniculum vulgare*)	Oil	843.88 (502.16–1458.19)	1736.30 (1230.01–3494.77)	1989.29 (1402.44–4106.03)	19.74 (0.001 ^a^)	Y = −1.04 + 1.24 × 10^−3X^
	Mg-LDH-F	451.95 (304.81–663.28)	880.32 (667.46–1439.86)	1001.75 (755.14–1675.14)	12.76 (0.003 ^a^)	Y = −1.2 + 1.70 × 10^−3X^
	Ni-LDH-F	550.12 (187.53–3382.85)	1116.31 (687.92–13,917)	1276.82 (786.49–16,976.02)	34.96 (0.000 ^a^)	Y = −1.12 + 1.68 × 10^−3X^
Green tea (*Camellia sinensis*)	Oil	938.93 (634.38–1453.09)	1944.13 (1365.39–3174.64)	2100.75 (1547.09–3688.21)	15.15 (0.004 ^a^)	Y = −1.2 + 1.76 × 10^−3X^
	Mg-LDH-GT	530.46 (173.93–1416.95)	1130.82 (729.36–4632.54)	1301.02 (836.27–5594.66)	31.95 (0.000 ^a^)	Y = −1.2 + 1.76 × 10^−3X^
	Ni-LDH-GT	769.94 (432.83–1362.39)	1633.88 (1145.95–3407.41)	1878.79 (1311.81–4023.44)	20.20 (0.000 ^a^)	Y = −1.2 + 1.76 × 10^−3X^

* LC_50_ values = ppm. The superscript a indicates that the differences between the groups are significant (by one-way ANOVA, Tukey’s range test, *p* < 0.05).

**Table 5:** The adulticidal effects of fennel oil and its LDH against adult *Culex pipiens,* 24 h post treatments.

Oil Name	Nanocomposite	Conc. (%)	Mortality% (Mean ± SE)	LC_50_ (Low.–Up.)	LC_90_ (Low.–Up.)	LC_95_ (Low.–Up.)	Chi (Sig)	Equation
Fennel (*Foeniculum vulgare*)	Oil	0	0.00 ± 0.00 ^c^	3.25 (2.00–4.35)	34.72 (21.80–80.40)	72.40 (38.76–230.68)	26.52 (0.000 ^a^)	Y = 0.64 + 0.6 × 10^−3X^
		2.0	10.00 ± 11.55 ^d^					
		5.0	53.33 ± 3.33 ^d^					
		10.0	66.67 ± 8.82 ^c^					
		15.0	76.67 ± 3.33 ^b^					
		20.0	86.67 ± 3.33 ^a^					
	Mg-LDH-F	0.0	0.00 ± 0.00 ^c^	1.75 (0.55–2.15)	8.52 (5.80–20.4)	14.16 (8.40–28.92)	18.904 (0.001 ^a^)	Y = −1.04 + 1.24 × 10^−3X^
		0.5	66.67 ± 6.67 ^d^					
		1.0	70.00 ± 5.77 ^c^					
		2.0	83.33 ± 8.82 ^b^					
		3.0	96.67 ± 3.33 ^a^					
		4.0	100.00 ± 0.00 ^a^					
	Ni-LDH-F	0.0	0.00 ± 0.00 ^c^	2.30 (1.35–3.145)	17.52 (12.72–29.52)	33.16 (21.36–70.68)	26.52 (0.000 ^a^)	Y = 0.64 + 0.6 × 10^−3X^
		0.5	56.67 ± 3.33 ^e^					
		1.0	63.33 ± 3.33 ^d^					
		2.0	73.33 ± 6.67 ^c^					
		3.0	86.67 ± 3.33 ^b^					
		4.0	93.33 ± 3.33 ^a^					

The different superscript letters indicate that the differences between the groups are significant by (one-way ANOVA, Tukey’s range test, *p* < 0.05).

**Table 6:** The adulticidal effects of green tea oil and its LDH against adult *Culex pipiens*, 24 h post treatments.

Oil Name	Nanocomposite	Conc. (%)	Mortality% (Mean ± SE)	LC_50_ (Low.–Up.)	LC_90_ (Low.–Up.)	LC_95_ (Low.–Up.)	Chi Sig)	Equation
Green tea (*Camellia sinensis*)	Oil	0.0	0.00 ± 0.00 ^e^	5.45 (3.95–6.95)	25.90 (18.83–47.45)	35.39 (22.04–202.28)	26.52 (0.000 ^a^)	Y = 1.10 + 0.17 × 10^−3X^
		2.0	40.00 ± 5.77 ^d^					
		5.0	43.33 ± 3.33 ^d^					
		10.0	56.67 ± 3.33 ^c^					
		15.0	70.00 ± 5.77 ^b^					
		20.0	76.67 ± 3.33 ^a^					
	Mg-LDH-GT	0.0	0.00 ± 0.00 ^f^	0.42 (3.27–6.83)	4.51 (2.30–7.99)	8.84 (5.51–20.57)	18.904 (0.001 ^a^)	Y = −0.25 + 1.2 × 10^−3X^
		0.5	56.67 ± 3.33 ^e^					
		1.0	66.67 ± 6.67 ^d^					
		2.0	76.67 ± 3.33 ^c^					
		3.0	83.33 ± 3.33 ^b^					
		4.0	93.33 ± 3.33 ^a^					
	Ni-LDH-GT	0.0	0.00 ± 0.00 ^f^	0.45 (031–0.79)	12.32 (6.76–41.91)	29.14 (12.90–159.03)	16.35 (0.001 ^a^)	Y = −0.97 + 0.17 × 10^−3X^
		0.5	50.00 ± 0.00 ^e^					
		1.0	56.67 ± 3.33 ^d^					
		2.0	66.67 ± 3.33 ^c^					
		3.0	73.33 ± 3.33 ^b^					
		4.0	83.33 ± 3.33 ^a^					

The different superscript letters indicate that the differences between the groups are significant by (one-way. ANOVA, Tukey’s range test, *p* < 0.05).

**Table 7:** Field evaluation of the applied materials against adult mosquito in different homes, 24 post-treatment.

Oil Name	Oil Formulated	Sites	Avg. Adult/Site	Mean Number ± SE	% Reduction	% Mean Reduction
	Control	home 1	13.7	22.36 ± 5.92 ^a^	0	0
		home 2	19.7		0	
		home 3	33.7		0	
Fennel (*Foeniculum vulgare*)	Oil	home 1	2.67	6.66 ± 1.38 ^b^	84.6	83.1
		home 2	5.00		81.2	
		home 3	7.33		83.6	
	Mg-LDH	home 1	0.00	0.22 ± 0.22 ^b^	100.0	100.0
		home 2	0.00		100.0	
		home 3	0.00		100.0	
Green tea (*Camellia sinensis*)	Oil	home 1	4.00	5.0 ± 1.34 ^b^	77.3	77.0
		home 2	7.33		72.8	
		home 3	8.67		80.8	
	Mg-LDH	home 1	0.00	0.00 ± 0.00 ^b^	98.3	99.0
		home 2	0.00		100.0	
		home 3	0.67		98.6	

Numbers of the same column followed by the same small letter are not significantly different (one-way ANOVA, Tukey’s range test, *p* > 0.05).

**Table 8:** The mean number of mosquito larvae, *Culex pipiens* consumed by some predators before and after being treated with fennel and green tea oil, and their Mg-LDH under laboratory conditions.

Oil Name	Treatment	Mosquito Predator Types			Control ***	Mean of Predation
		*G. affinis*	*C. tripunctatus*	*S. urinator*		
*Foeniculum vulgare*	Free- Mg-LDH *	80.00 ± 1.15 ^aB^	64.33 ± 4.18 ^bC^	25.00 ± 0.58 ^bC^	99.67 ± 0.33 ^aA^	67.25 ± 8.32 ^a^
	Oil	74.67 ± 2.03 ^bB^	52.33 ± 2.03 ^cC^	22.33 ± 1.76 ^cC^	99.33 ± 0.67 ^aA^	62.17 ± 8.59 ^b^
	Mg-LDH-F	75.00 ± 1.15 ^bB^	69.00 ± 3.21 ^aC^	27.33 ± 2.19 ^aC^	99.00 ± 0.58 ^aA^	67.58 ± 7.83 ^a^
*Camellia sinensis*	Free Mg-LDH **	82.33 ± 1.15 ^bB^	65.00 ± 0.00 ^bC^	25.00 ± 0.58 ^bC^	97.67 ± 0.33 ^aA^	67.50 ± 6.32 ^a^
	Oil	75.67 ± 1.76 ^cB^	55.67 ± 1.20 ^cC^	23.33 ± 2.03 ^bC^	99.33 ± 0.67 ^aA^	63.50 ± 8.43 ^b^
	Mg-LDH-GT	77.00 ± 3.21 ^cB^	71.00 ± 1.73 ^aC^	28.00 ± 1.53 ^aC^	99.00 ± 0.58 ^aA^	68.75 ± 7.81 ^a^
Mean of sample		77.05 ± 0.94 ^B^	62.77 ± 1.72 ^C^	25.15 ± 0.65 ^C^		99.33 ± 0.19 ^A^

a, b and c: There is no significant difference (*p* > 0.05) between any two means, within the same column that have the same superscript letter. A, B and C: There is no significant difference (*p* > 0.05) between any two means for the same attribute, within the same row that have the same superscript letter. * Control with free Mg- LDH-F, ** control with free Mg- LDH-GT, *** control without treatment.

## Data Availability

All data analyzed during this study are included in this published article.

## Sample Availability

Samples of the compounds are not available from the authors.
